# CD8^+^ T Cells Mediate Robust Stage-Specific Immunity to *P. berghei* under Chemoprophylaxis and This Protective Environment Is Not Downregulated by the Presence of Blood-Stage Infection

**DOI:** 10.1371/journal.pone.0088117

**Published:** 2014-02-07

**Authors:** Matthew D. Lewis, Johannes Pfeil, Kirsten Heiss, Ann-Kristin Mueller

**Affiliations:** 1 Department of Infectious Diseases, Parasitology Unit, University Hospital Heidelberg, Heidelberg, Germany; 2 Centre for Childhood and Adolescence Medicine, University Hospital Heidelberg, Heidelberg, Germany; Institut de Recherche pour le Développement, France

## Abstract

Sterile protection against malaria infection can be achieved by the inoculation of intact sporozoites while treating concomitantly with the 4-aminoquinoline chloroquine. We present an analysis of protective immunity elicited by successive immunization with *Plasmodium berghei* sporozoites under chemoprophylaxis. Immunization resulted in a protective, stage-specific immune response. Protection appeared to be mediated by CD8^+^ T cells and was abrogated upon their specific depletion. Adoptive transfer of splenocytes rendered recipient animals resistant to sporozoite infection, but not to blood-stage challenge. Immunization with sporozoites under chemoprophylaxis results in robust immunity, and the presence of blood-stage infection at sporozoite immunization had no downregulating effect on the protective immune response.

## Introduction

Malaria is a devastating disease with an annual death toll of up to one million people [1]. Immune responses caused by the infections are only partly understood [2] and a vaccine is still elusive.

Studies amongst residents of malaria endemic areas indicate that non-sterile immunity mostly targets the blood stage and only develops gradually after multiple exposures [3], [4]. The resulting semi-immunity protects individuals against severe manifestations of the disease but is said to decay rapidly once exposure to the parasite ceases [2].

In contrast to the situation in endemic regions, multiple experimental vaccine studies [5], [6], [7], [8] have shown that immunization with attenuated whole sporozoites is capable of generating sterilizing pre-erythrocytic protection against subsequent experimental sporozoite challenge [9]. To achieve this, parasites used for vaccination are attenuated during their pre-erythrocytic development. Attenuation is achieved either by gamma-irradiation [5], [10] of infectious sporozoites or specific deletion of genes critical for liver-stage development [6], [11], [12].

An alternative option is to inoculate mice with wildtype sporozoites under prophylactic drug cover, using an anti-erythrocytic antimalarial compound. Several drugs have been tested in this approach; however most studies utilize chloroquine (CQ). CQ is an antimalarial drug that kills parasites within red blood cells, accumulating at high concentrations within the blood-stage digestive vacuole [13]. It forms non-covalent complexes with heme [14], interferes with heme sequestration to the less toxic product hemozoin and poisons the parasite via the accretion of such drug-heme complexes [15]. Since the onset of clinical symptoms occurs with the infection of erythrocytes, CQ can be administered prophylactically so that the parasite infection manifests within the liver-stage phase but does not develop to blood-stage infection and clinical pathology [9].

This form of immunization, termed chloroquine chemoprophylaxis (CPS), thus permits complete pre-erythrocytic development of the parasite prior to its destruction by the compound upon egress of merosomes from the liver into the blood [16]. Infection and the acquisition of immunity are thereby the result of experimental sporozoite wildtype infection, as opposed to the attenuated infection induced by genetically modified or radiation exposed parasites.

It is known that immunization of rodents with *Plasmodium yoelii* sporozoites under continuous CQ administration generates strong pre-erythrocytic immunity to subsequent wildtype challenge and to a lesser extent, erythrocytic immunity [17] comparable to radiation and genetically attenuated parasites [8], [17]. Recently, protective immunity to *P. falciparum* by chloroquine prophylaxis was generated in humans, and was correlated with the induction of parasite-specific effector memory T cells producing IFN-γ, TNF-α and IL-2 [7]. Protection is mediated by both CD8^+^ and CD4^+^ T cells, Nitric Oxide (NO) and IFN-γ [17]. A recent study using *P. berghei* found long-term responses that are mediated by CD8^+^ IFN-γ producing hepatic memory T cells [16].

The liver-stage directed cellular immune response induced by *Plasmodium* infection is only partly understood and it remains unclear why multiple infections in the field are incapable to generate a protective immune response comparable to experimental whole parasite vaccination. It has been suggested that *Plasmodium* blood stages may have a down-regulating influence upon the acquisition of pre-erythrocytic immunity in rodent models [18], [19], which could account for the absence of pre-erythrocytic immunity developing in endemic areas. Within this study, we assessed CPS-induced pre-erythrocytic immunity in the *P. berghei* and C57BL/6 mouse malaria model. We identified the underlying cellular immunity and investigated the transferability of this protective immunity. We finally demonstrate the robustness of the immune response, as CPS vaccination under ongoing blood-stage infection still resulted in protective liver-stage directed immunity. Downregulation of pre-erythrocytic immunity by blood-stage infection is a popular hypothesis in the literature, which may partially explain why individuals living in endemic areas do not develop adequately protective pre-erythrocytic immune responses. This manuscript presents, by minimal alteration of the established protocol, an attempt to test this theory in the CPS model.

## Materials and Methods

### Ethics Statement

All animal experiments were performed according to European regulations concerning FELASA category B and GV-SOLAS standard guidelines. Animal experiments were approved by the German authorities (Regierungspräsidium Karlsruhe, Germany), § 8 Abs. 1 Tierschutzgesetz (TierSchG) under the license G-6/09 (“MALBI: Malaria Biologie und Immunologie”/”The biology and immunology of malaria”).

### Parasite, Mice and Chloroquine Prophylaxis

For all experiments, C57BL/6 mice were purchased from Charles River Laboratories (Sulzfeld, Germany) and kept under specified pathogen-free (SPF) conditions within the animal facility at Heidelberg University (IBF). 6-week old female C57BL/6 mice were administered oral chloroquine dissolved in tap water as described previously [21]. At a consumption of 4–6 ml per day, 0.29 mg/ml chloroquine diphosphate salt dissolved in water renders administration of 1.2–1.8 mg/day. For parasite infection experiments *P. berghei* NK65 [20] sporozoites were extracted from salivary glands of infected *An. stephensi* mosquitoes. 10,000 sporozoites or 1×10^6^ *P. berghei* NK65 parasitized red blood cells (pRBC) were injected intravenously into mice. The presence of blood-stage parasites was determined by daily examination of Giemsa-stained blood smears.

### Immunofluorescence of Intrahepatic Malaria Stages

Human hepatoma cells (HuH7) [21] were cultivated in Dulbecco’s modified Eagle medium (Invitrogen) supplemented with 10% FCS and 1% Penicillin/Streptomycin (Invitrogen) at 37°C and 5% CO_2_. HuH7 cells were kindly provided by Ralf Bartenschlager, Department for Infectious Diseases, University Hospital Heidelberg, Heidelberg, Germany. To follow up intrahepatic development of *P. berghei* NK65, 30,000 HuH7 cells were seeded in 8-well chamber slides (Lab-Tek, NUNC) one day before the inoculation of 10,000 salivary gland-derived infectious sporozoites. Sporozoites were allowed to invade for 90 minutes and subsequently subjected to chloroquine exposure at a final inhibitory concentration of 10 µM. Liver-stage development was stopped at 48 h after infection by applying ice-cold Methanol (AppliChem) to the infected wells, followed by staining of parasitic HSP-70 protein [22] with anti-mouse Alexa Fluor 488 as secondary antibody (Molecular Probes). Evaluating the effect of antimalarial chloroquine treatment on liver-stage development was carried out by counting total liver-stage numbers using a Zeiss fluorescence microscope. Zeiss Image Examiner software was applied for measurement of liver-stages sizes. Statistics calculated by student’s *t* test. Asterisks signify degrees of significance where *** indicates P<0.001, ** indicates P = <0.01, * indicates P<0.05 and “ns” indicates P>0.05.

### Blood-stage Infection

Mice were immunized three times with 10,000 infectious sporozoites at days 0, 21, and 42. Four days prior to each sporozoite immunization, mice were infected with 1×10^6^ parasitized red blood cells (CPS-bs). In order to ensure any residual chloroquine was metabolized, chloroquine drinking water was taken off and replaced with normal water one week prior to pRBC administration. After pRBC infection, blood-stage parasitemia was determined by examination of Giemsa-stained blood smears. Blood stage infection was maintained for 4 days until sporozoite inoculation 4 days later. On day 4, after sporozoite inoculation, animals were re-administered chloroquine. Chloroquine treatment caused blood parasitemia to decrease to zero within 2 days, hence, inclusive of that period, animals were blood-stage positive for approximately 5 days.

### *In vivo* CD8^+^ T-cell Depletion Experiments

For depletion of CD8^+^ T cells, experimental mice were treated with rat anti-mouse CD8a (clone 53–6.7, Biolegend, USA) antibody. CD8 depleting antibody was administered by intraperitoneal injection of 150 µg/per mouse 3 days prior to challenge and every 3 days thereafter until the endpoint of the experiment [23]. Efficacy of CD8 T-cell depletion was confirmed by flow cytometry analysis by staining peripheral blood leukocytes from mice with an anti-mouse CD8b.2 (clone 53–5.8, BD Bioscience) antibody. These samples were found to be between 90–96% free of CD8^+^ T cells.

### Adoptive Cell-transfer Experiments

Spleens were removed, homogenized and subjected to erythrocyte lysis with erythrocyte lysis buffer (8.26 g NH_4_Cl, 1 g KHCO_3,_ 0.037 g EDTA in 1l ddH_2_O) at room temperature for 10 minutes. Isolated spleen cells were quantified and 2×10^7^ cells were injected intravenously as bulk splenocytes into naïve recipient mice. Recipient mice were challenged by intravenous inoculation of 10,000 sporozoites 24 hours after splenocyte transfer.

### Quantitative Real-time PCR (qrt-PCR)

Mice were sacrificed 46 to 48 hours post-infection, and livers were removed and homogenized using a TissueRuptor (Qiagen) in QIAzol reagent. Total RNA was isolated from QIAzol and complementary DNA (cDNA) was synthesized with the RETROScript kit (Ambion), both according to the manufacturers’ instructions. Quantitative RT-PCR was performed with the ABI 7500 sequence detection system with Power SYBR Green PCRMasterMix (Applied Biosystems), according to the manufacturer’s instructions. Relative liver parasite levels were quantified by determining the mean *Ct* value of the parasitic *18S ribosomal subunit* using gene-specific primers [GenInfo Identifier (GI), 160641] (forward: 5′-AAGCATTAAATAAAGCGAATACATCCTTAC-3′; reverse: 5′-GGAGATTGGTTTTGACGTTTATGTG-3′), normalized to the mean *Ct* mouse *GAPDH* values (GI, 281199965) (forward: 5′-CGTCCCGTAGACAAAATGGT-3′; reverse:5′-TTGATGGCAACAATCTCCAC-3′) using the ΔΔCt method where both CPS and CPS-bs data were normalized to infected but unvaccinated control mice (with a mean relative expression of 1). Expression levels of intrahepatic CD8 (forward: 5′-GGCTTCATCCCACAACAAGATAA-3′; reverse: 5′-TCCCTCATGGCAGAAAACAGTT-3′), TNF-α (forward: 5′- TGTGGCAGGGGCCACCACGC-3′; reverse: 5′- CCAGGAGGGCGTTGGCGCGC-3′) and IFN-γ (forward: 5′- AGGAACTGGCAAAAGGATGG-3′; reverse: 5′- CTGTGGGTTGTTGACCTCAA-3′) was additionally determined by ΔΔCt normalized to naïve uninfected mice. Temperature profile: 95°C for 15 min, and then 40 cycles of 95°C for 15 s, 55°C for 15 s and 60°C for 45 s.

### Statistical Analysis

Data assessment and statistical analysis was performed using Prism Version 5.0b (GraphPad Software, San Diego California, USA). Mann-Whitney-U-test, student’s t test was used to compare means of relative liver parasite levels and Log-rank (Mantel-Cox) test was used to compare survival distributions.

## Results and Discussion

### Chloroquine has no Effect on *P. berghei* Liver-stage Development *in* *vitro*

**Figure 1:**
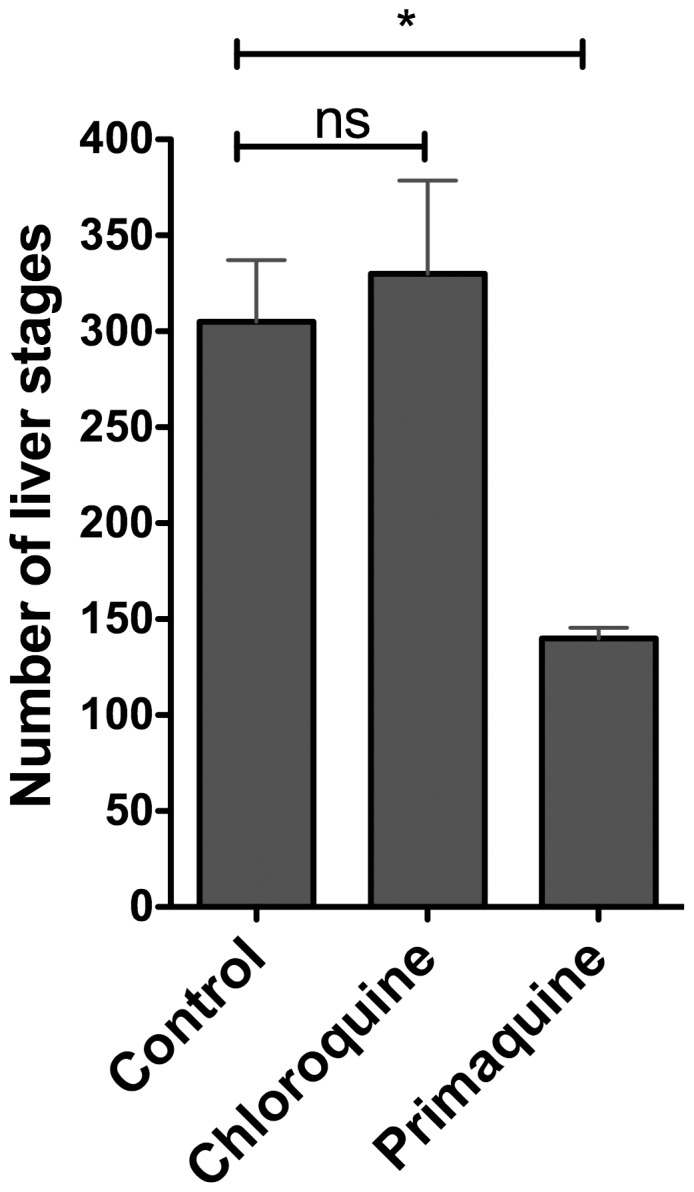
CQ has no effect on *Plasmodium berghei* liver-stage numbers *in* *vitro*. 10,000 *P. berghei* NK65 sporozoites were allowed to invade HuH7 cells and subsequently cultured in DMEM culture medium (with 10% FCS and 1% Penicillin/Streptomycin) alone or supplemented with 10 µM chloroquine or 10 µM primaquine. Parasites were permitted to develop over 48 hours before being fixed, stained with anti-HSP70 antibody [22] and counted. Numbers of liver stages per well were counted by fluorescent microscopy in duplicates. Error bars represent standard deviation. Statistics calculated by student’s *t* test. Asterisks signify degrees of significance where *** indicates P<0.001, ** indicates P = <0.01, * indicates P<0.05 and “ns” indicates P>0.05.

It has been previously demonstrated that CQ does not inhibit the emergence of blood stage parasites when applied during the liver-stage phase [24], [25] and it has been shown in *P. yoelii* that it does not impede parasite development in the liver [17]. To provide further proof-of-principle and to test this hypothesis in *P. berghei*, we determined the effect of CQ on *in* *vitro P. berghei* liver-stage development. *P. berghei* sporozoites were added to cultures of HuH7 human hepatoma cells, allowed to invade, and subsequently cell culture media was supplemented with CQ. Disturbance of liver-stage development due to CQ was assessed by measuring the number and morphology of intrahepatic parasites by immunofluorescence microscopy. As anticipated, liver-stage development in the presence of CQ showed no significant reduction in number of liver stages at 48 hours, compared to the untreated control. In contrast, the development of intrahepatic stages cultured in the presence of control substance primaquine, an 8-aminoquinoline with potent liver-stage activity [26], is significantly inhibited (Figure 1).

### Sporozoite-immunized C57BL/6 are Protected against Subsequent *P. berghei* NK65 Sporozoite Challenge

**Figure 2:**
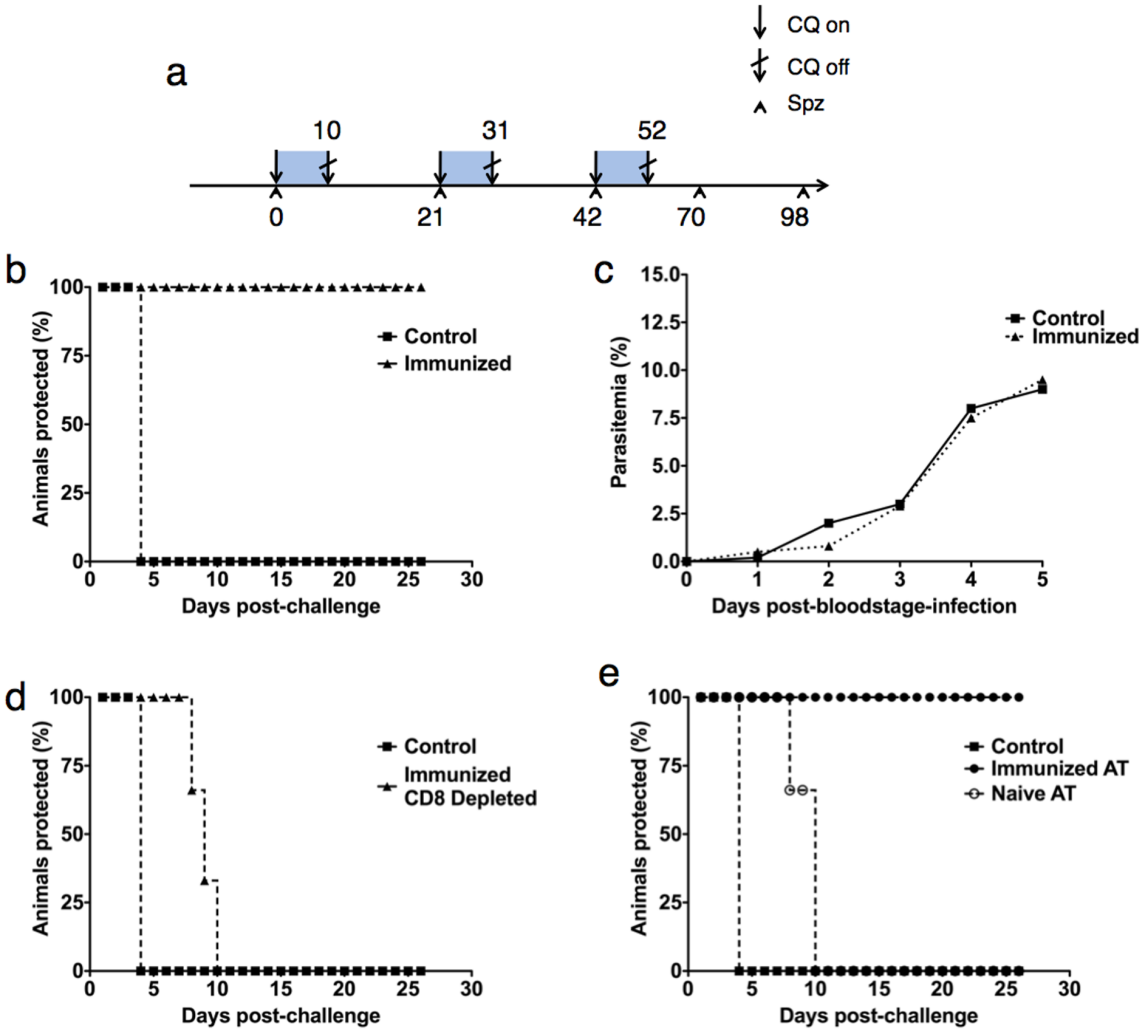
Figure 2a. Scheme to illustrate the CPS immunization regime. Animals were subjected to three immunization shots of 10,000 *Pb*NK65 sporozoites at days 0, 21 and 42. Immediately after each immunization animals were administered chloroquine *ad libitum* for 10 days. Blue blocks represent periods of time under chloroquine cover. Animals were subjected to challenge with 10,000 sporozoites at day 70 and, in some cases, rechallenged at day 98. **Figure 2b** **.** **CPS vaccination generates sterilizing immunity against subsequent** ***P. berghei*** **challenge.** Animals (n = 10) were subjected to CPS as described and subsequently challenged with 10,000 *Pb*NK65 sporozoites at day 70 post-first-immunization. Naïve age-matched animals in the Control group were challenged with 10,000 sporozoites only. Animals were monitored for presence of blood stage parasitemia via Giemsa staining of daily blood smears. **Figure 2c** **. CPS vaccination does not protect against blood stage challenge.** Animals (n = 5) were subjected to CPS as described and subsequently challenged with 1×10^6^ parasitized erythrocytes at day 70 post-first-immunization, 28 days post-third-immunization. Naïve age-matched animals in the Control group were challenged with 1×10^6^ parasitized erythrocytes only. Animals were monitored for presence of blood-stage parasitemia via Giemsa staining of daily blood smears. **Figure 2d** **. CD8^+^ T cells are essential for sterilizing immunity elicited by CPS vaccination.** Animals (n = 6) were subjected to CPS as described, subsequently depleted of their CD8^+^ T cells via administration of neutralizing antibody and subsequently challenged with 10,000 sporozoites at day 70 post-first-immunization, 28 days post-third-immunization. Naïve age-matched animals in the Control group were challenged with 10,000 sporozoites only. Animals were monitored for presence of blood-stage parasitemia via daily staining of Giemsa blood smears. Comparison of curves P = 0.0009 by Log-rank (Mantel-Cox) Test. **Figure 2e** **. Protective immunity can be transferred to naïve animals by adoptive transfer of splenocytes.** Animals (n = 6) were subjected to CPS as described. CPS immunized animals were sacrificed and their splenic lymphocytes isolated and purified and 2×10^7^ transferred into a group of naïve recipient animals, “Immunized AT”. Control recipient animals, “Naïve AT”, received splenocytes from naïve donors. All experimental groups, including a naïve group were challenged with 10,000 sporozoites 24 hours later. Animals were monitored for presence of blood-stage parasitemia via daily staining of Giemsa blood smears. Comparison of “Immunized AT” and “Naïve AT” curves P = 0.025 by Log-rank (Mantel-Cox) Test. Comparison of all three curves P = 0.091 by Log-rank (Mantel-Cox) Test.

Previous rodent studies applying continuous CQ treatment have used *P. yoelii* and daily intraperitoneal injection of CQ in order to achieve sterilizing immunity to subsequent wild-type sporozoite challenge [17], [27]. Our study applied an altered immunization system, based on *ad libitum* administration of CQ in the drinking water [28]. In order to demonstrate that CPS-induced sterile immunity can be generated to *P. berghei* NK65 (*Pb*NK65) challenge via *ad libitum* drinking water administration, we first assessed the protective capacity of an immunization schedule where animals were subjected to three intravenous sporozoite immunizations (Figure 2a). Mice were kept under intermittent CQ drinking water supply starting from day 0 to week 8.5 (day 52 post-first-immunization) and were immunized by intravenous administration of 10,000 infectious sporozoites at weeks 0, 3 and 6 (days 0, 21 and 42 post-first-immunization).

Immunized animals were subjected to challenge with 10,000 *Pb*NK65 sporozoites intravenously (i.v.) at week 10 (70 days post-first-immunization), 18 days after CPS was stopped. Experimental animals subjected to CPS remained blood-stage negative by microscopic examination of Giemsa-stained blood smears up to four weeks post-challenge (14 weeks post-first-immunization) whereas control mice that underwent CPS and were subjected to sporozoite challenge in the absence of prior sporozoite immunization became blood-stage positive (Figure 2b). Mice were re-challenged 4 weeks after challenge at week 14 post-first-immunization (day 98 post-first-immunization) by intravenous injection of 10,000 *Pb*NK65 sporozoites and remained blood-stage negative (data not shown). This indicates the capacity of the applied *Pb*NK65 sporozoite immunization schedule to generate protective immune responses (Figure 2b).

### CPS-induced Immunity is Stage-specific

A previous study in *P. yoelii* [14] demonstrated that sporozoite immunization under CPS elicits a protective response, albeit minor, towards the parasite at the blood stage in addition to the liver. To determine if there is cross-stage specificity in the protection elicited by CPS, immunized mice were challenged with 1×10^6^ parasitized erythrocytes at day 70 post-first-immunization. Mice remained susceptible to challenge by parasitized red blood cells (pRBC), with a parasitemia growth curve indistinguishable to blood stage infection in malaria naïve mice. At the given threshold of blood stage inoculum, we found that the protective immunity elicited by CPS is conserved to the liver stage and unable to exert any protective effect against symptomatic blood stage infection. Parasitemia reached approximately 10% at day 5 post-infection and continued without notable deviation from the naïve-challenged control (Figure 2c).

### CD8^+^ T Cells are Required for Protection against Sporozoite Challenge in Immunized Mice

The protective immune response caused by immunization under CPS has not been extensively studied, although it is thought to be mediated by CD8^+^ T cell responses and IFN-γ targeting infected hepatocytes [29]. To test this hypothesis, immunized mice were depleted of their CD8^+^ T cells via injection of anti-CD8 neutralizing antibodies prior to challenge with 10,000 *Pb*NK65 sporozoites. Depletion of CD8^+^ T cells rendered all mice susceptible to malaria infection, indicating that CD8^+^ T cells are a necessary prerequisite for protection (Figure 2d). Interestingly, the onset of patency in CD8^+^ T cell-depleted mice was delayed by several days compared to naïve controls. This suggests that a degree of protective immunity remained that, while not sufficient to prevent the onset of blood patency, is able to delay either pre-erythrocytic maturation or early blood stage infection. This protection is comparable to the delay in patency observable in *RAG1* ^−/−^ mice (lacking mature B and T lymphocytes), immunized with genetically attenuated parasites [30] and may indicate a limited role for other components of the immune system, such as players of the innate immunity e.g. NK cells, or perhaps CD4^+^ T cells, in the protection induced by attenuated pre-erythrocytic models.

### Transfer of Splenocytes from Immunized Animals into Naïve mice Results in Protection against Infectious Sporozoite Challenge

Given that protection was mediated by a CD8^+^ T cell immune response, we further questioned whether CD8^+^ T cells from immunized mice are able to protect naïve recipient mice against sporozoite challenge. Naïve animals were injected intravenously with 2×10^7^ splenocytes from immunized animals and subjected to challenge with 10,000 wildtype sporozoites 24 hours later. Recipient animals remained protected, confirmed by Giemsa-stained blood smears remaining negative for blood parasitemia, whereas control animals that received splenocytes isolated from naïve donors developed blood-stage infection (Figure 2e).

Given that *in vivo* neutralization of CD8^+^ T cells rendered the mice susceptible to subsequent sporozoite challenge, and transfer of whole splenocytes protected naïve animals against sporozoite challenge, our data indicate that the protective immune response is CD8^+^ T cell-derived and entirely transferable between hosts. Thus, our experiments further corroborate the limited existing data that suggests CD8^+^ T-cell as the mediating protective immune mechanism in CPS vaccination [16], [17]. Our data support previous findings that sporozoite infection combined with chloroquine chemoprophylaxis (CPS), as a controlled experimental system, is a potent inducer of liver-directed immunity, with parasite clearance directed sparsely or not at all towards the blood stage [16], [31].

### Protection is Robust and Resistant to Blood Stage Influence

It is not clear why natural infection only results in a relatively weak degree of pre-erythrocytic protection as compared to experimental vaccination. It has been suggested [7], [18] that the presence of *Plasmodium* blood stages has a down-regulating influence upon the acquisition of pre-erythrocytic immunity in rodent models [27], [32]. Indeed, a previous study in the *P. berghei* model showed that mice permitted to develop high blood-stage parasitemias which were then subsequently treated with chloroquine resulted in incomplete protection to *P.berghei* sporozoite immunization [19]. It was then demonstrated that blood-stage suppression of liver-stage immunity might be mediated by blood-stage induced dendritic cells suppressing CD8^+^ T-cell responses [18]. More recently, it was shown that the presence of blood-stage parasitemia in malaria superinfection directly inhibits the development of subsequent liver stage infection as the result of increased production of iron-regulatory hormone hepcidin [33].

**Figure 3:**
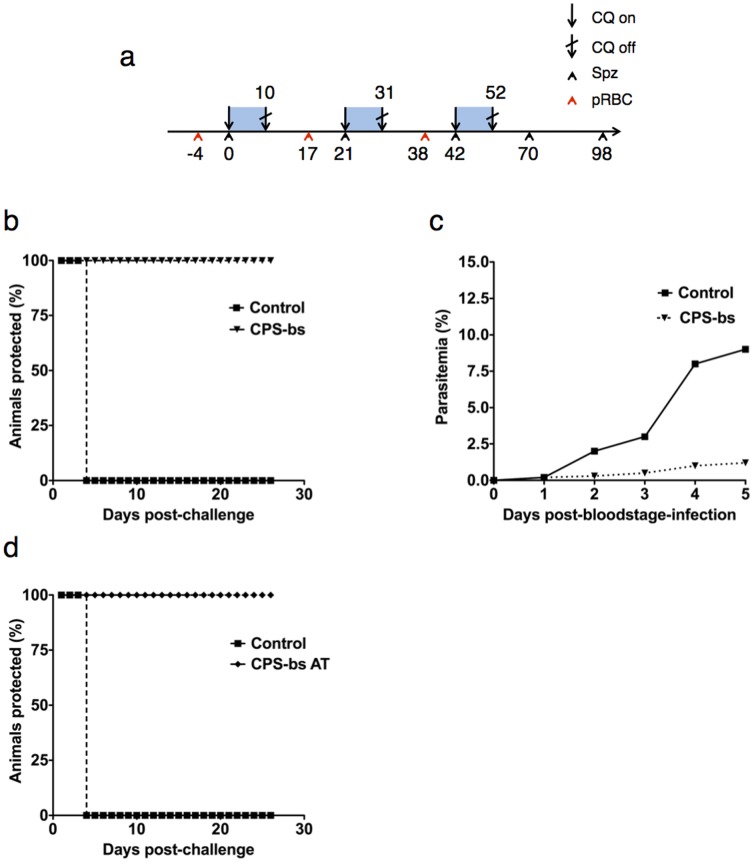
Figure 3a.Scheme to illustrate the CPS-bs immunization regime. Animals were subjected to three immunization shots of 10,000 *Pb*NK65 sporozoites at days 0, 21 and 42. Immediately after each immunization animals were administered chloroquine *ad libitum* for 10 days. Blue blocks represent periods of time under chloroquine cover. Animals were subjected to challenge with either 1×10^6^ pRBC or 10,000 sporozoites at day 70 and, in some cases, rechallenged at day 98. Those animals in the CPS-bs groups were subjected to infection with 1×10^6^ pRBC 4 days prior to each sporozoite immunization, at days −4, 17 and 38 as indicated. **Figure 3b** **. CPS vaccination in patent animals generates sterilizing immunity with no apparent downregulation of immunity.** Animals (n = 5) were subjected to CPS under patency (CPS-bs) as described and subsequently challenged with 10,000 NK65 sporozoites at day 70 post-first-immunization. Naïve age-matched animals in the Control group were challenged with 10,000 NK65 sporozoites only. Animals were monitored for presence of blood-stage parasitemia via Giemsa staining of daily blood smears. **Figure 3c** **. CPS-bs vaccination partially inhibits blood-stage growth but does not protect against blood-stage challenge.** Animals (n = 5) were subjected to CPS under patency (CPS-bs) as described and subsequently challenged with 1×10^6^ parasitized erythrocytes. Naïve age-matched animals in the Control group were challenged with 1×10^6^ parasitized erythrocytes only. Animals were monitored for presence of blood stage parasitemia via Giemsa staining of daily blood smears. **Figure 3d** **. Protective immunity can be transferred by adoptive transfer with no apparent downregulation of CPS immunity.** Animals (n = 5) were subjected to CPS under patency as described. CPS-bs animals were sacrificed and their splenic lymphocytes isolated and purified and transferred into naïve recipient animals, “CPS-bs AT”. Age-matched animals in the control group were naïve. All mice were subsequently challenged with 10,000 *Pb*NK65 sporozoites 24 hours later. Animals were monitored for presence of blood-stage parasitemia via daily staining of Giemsa blood smears.

**Figure 4:**
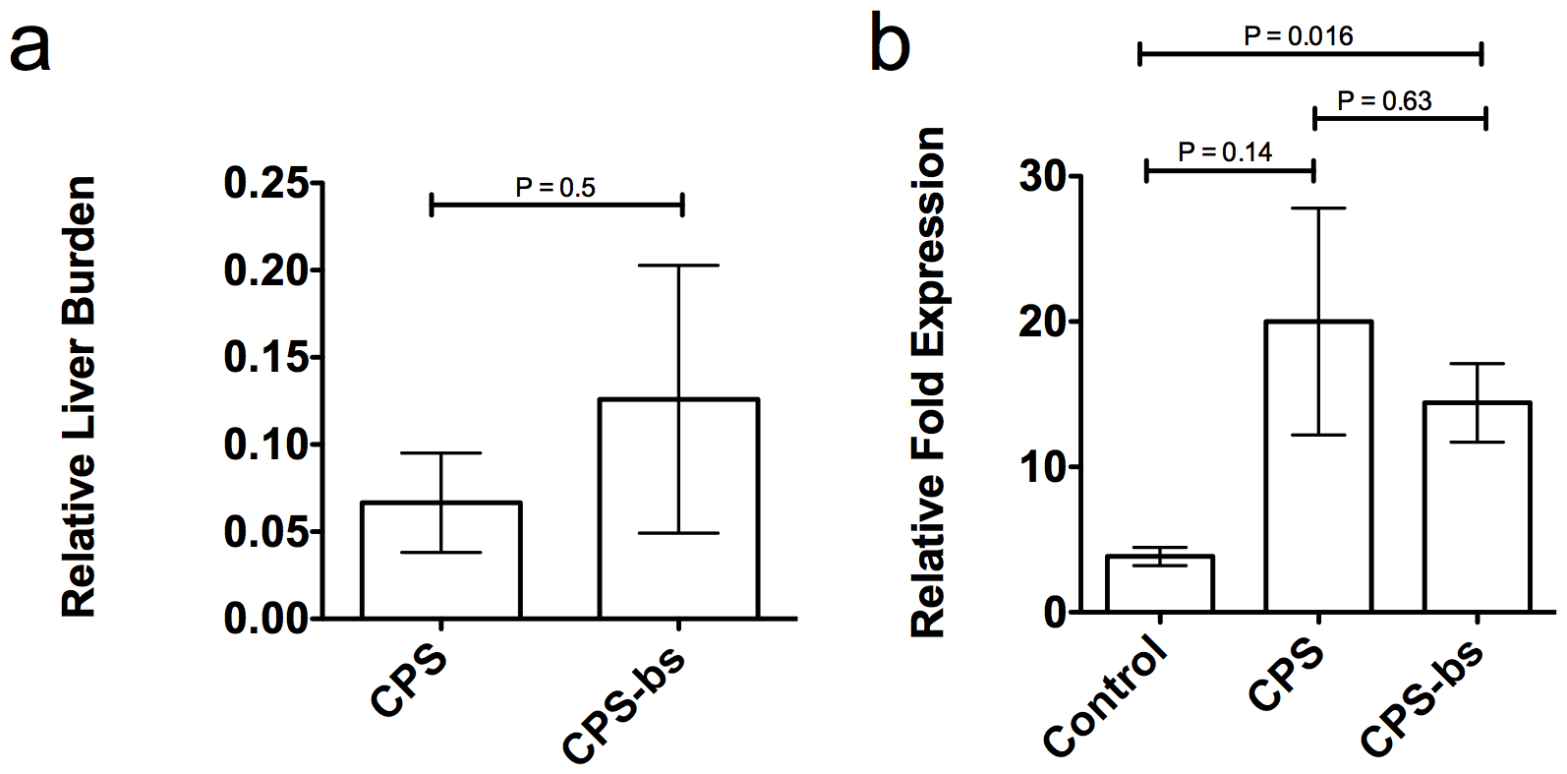
Figure 4a. Parasite liver load is not significantly different between CPS and CPS-bs groups. Animals (n = 3) were subjected to CPS (CPS) or CPS under patency (CPS-bs) as described and subsequently challenged with 10,000 NK65 sporozoites at day 70 post-first-immunization. Mice were sacrificed after 46–48 hours and parasite liver burden was quantified by quantitative real-time PCR analysis of parasitic 18S rRNA expression and normalized to infected but unvaccinated control mice. **Figure 4b** **. Both normal CPS and CPS-bs induce increased CD8 expression in the liver.** Animals (n = 4) were subjected to CPS (CPS) and CPS under patency (CPS-bs) as described and subsequently challenged with 10,000 NK65 sporozoites at day 70 post-first-immunization. Unvaccinated control mice (Control) were infected with 10,000 NK65 sporozoites. Mice were sacrificed after 46–48 hours and expression of CD8 was quantified by quantitative real-time PCR analysis.

Taking this into consideration, we tested whether the presence of blood-stage infection at the time of liver-stage development may downregulate the acquisition of pre-erythrocytic immunity in vaccination under chemoprophylaxis. To achieve this, we inoculated mice with 1×10^6^ parasitized erythrocytes three days prior to each immunization with 10,000 sporozoites to generate a model whereby blood stage parasitemia is present at the time of sporozoite administration (CPS-bs immunization, Figure 3a) in order to assess possible effects of blood-stage parasitemia on the acquisition of pre-erythrocytic immunity. This modified immunization schedule (CPS-bs), whereby mice received repeated sporozoite immunizations under the presence of blood stage parasitemia, resulted in full protection against subsequent sporozoite challenge, comparable to the observed phenotype in the CPS-immunization protocol (Figure 3b). We additionally found that the modified immunization schedule (CPS-bs) resulted in reduced growth of blood stage parasites as compared to naïve mice or mice receiving CPS alone. This is indicative of an immune response, likely antibody-mediated, targeting the parasite at the blood stage that, while incapable of eliminating the infection, is sufficient to stunt its normal erythrocytic growth. However, this blood-stage directed immune response was incapable of eliminating blood-stage malaria infection (Figure 3c). Transfer of splenocytes from modified-immunized donors (CPS-bs) to naïve recipient animals resulted in protection against subsequent sporozoite challenge (Figure 3d). Thus, sufficient cell-mediated immune responses were generated and presence of blood-stage parasites does not have a clinically significant down-regulating effect on the development of liver-stage immunity. Parasite liver load measured by qRT-PCR was not statistically different in the CPS-bs group relative to the regular CPS group (Figure 4a). Furthermore, the surface marker CD8 was significantly upregulated in the livers of both vaccinated groups compared to a control group, as measured by quantitative mRNA transcription, suggesting a potentially higher number of CD8^+^ T cells present in the organ. There was no significant difference between the two vaccinated experimental groups (Fig 4b). We did observe a reduced level of TNF-α mRNA in the liver of animals subjected to the standard CPS regime, relative to control animals and those subjected to CPS-bs (Figure S1). There was no significant difference in IFN-γ mRNA expression. Our study addresses several gaps that exist in ongoing CPS studies, describes the protective immune responses induced by CPS and addresses several questions with regard to the absence of protective pre-erythrocytic immune responses in endemic regions. We present an analysis of the *P. berghei* CPS model by *ad libitum* administration of chloroquine, where we can clearly demonstrate that it succeeds in provoking robust pre-erythrocytic immune responses, which are specifically conserved to the liver stage. These responses are mediated by CD8^+^ T cells and can be transferred to naïve donor mice by adoptive transfer of whole splenocytes. Our data supports a recent publication in which humans subjected to sporozoite immunization under CPS did not develop any protection against blood-stage challenge [31]. Similarly, experimental animals in our study did not demonstrate any protection against blood-stage challenge in the CPS model, and limited parasite inhibition in CPS-bs. Significantly, we show that the pre-erythrocytic protective immune response is robust and effective even in the presence of a pre-existing blood-stage infection. To our knowledge, this is the first study that has addressed the role of blood-stage infection in downregulating or modulating the pre-erythrocytic immune response to malaria as generated by CPS vaccination. A previous study has addressed a similar question but did not use a three-shot-immunization regime under CPS [19]. In our analysis, CPS-bs and CPS immunization equally resulted in fully protective, liver stage-directed immune responses. In our experimental setup, by administration of 10^6^ infected red blood cells 4 days prior to sporozoite challenge, blood stage infection was present at the time point of sporozoite administration. This emulates the situation for individuals living in malaria endemic regions that have persisting parasitemia while still being exposed to infectious mosquito bites. Nonetheless, the methodology in this study does not provide an authentic reproduction of the situation in the field, which might involve the administration of chloroquine *following* the onset of blood stage parasitemia. The induction of an erythrocytic infection by sporozoite infection would involve different immune responses to those induced by infected erythrocytes, in particular in the liver and in the dermis [29], [34] and include a variety of immune responses at different stages of the parasite’s life cycle. It might be interesting to investigate the influence of an already passed intrahepatic and established intra-erythrocytic infection, by infectious mosquito bite inoculation, on the later acquisition of pre-erythrocytic immune responses induced by CPS. This would permit analysis of the numerous immune responses that occur at the interface between liver and blood stages. It is feasible that such responses would have a modulating effect on the pre-erythrocytic immunity.

Thus, we concede the possibility that blood stage infection as seen during natural exposure may have a suppressive effect on the development of long-lasting protective immune responses against liver stages. However, we avoided profound alteration of the CPS regime in this study and endeavored to influence the protective immunity afforded by vaccination under chloroquine chemoprophylaxis by minimal alteration of the already established protocol. The data from this study indicate that blood-stage infection does not have any influence on the pre-erythrocytic CD8^+^ T-cell-responses that protect against the parasite liver stage. We believe that our study provides the basis for further studies into investigating the protective immune responses generated by CPS and poses questions about the precise role of erythrocytic infection on pre-erythrocytic immunity.

## Supporting Information

Figure S1 **Cytokine expression in both CPS and CPS-bs groups.** Animals (n = 4) were subjected to CPS (CPS) or CPS under patency (CPS-bs) as described and subsequently challenged with 10,000 NK65 sporozoites at day 70 post-first-immunization. Unvaccinated control mice (Control) were infected with 10,000 NK65 sporozoites. Mice were sacrificed after 46–48 hours and TNF-α and IFN-γ mRNA quantified by quantitative real-time PCR analysis of cytokine mRNA expression.
(TIF)

## References

[1] Murray CJ, Rosenfeld LC, Lim SS, Andrews KG, Foreman KJ, et al (2012) Global malaria mortality between 1980 and 2010: a systematic analysis. Lancet 379: 413–431. 22305225 10.1016/S0140-6736(12)60034-8

[2] Langhorne J, Ndungu FM, Sponaas AM, Marsh K (2008) Immunity to malaria: more questions than answers. Nat Immunol 9: 725–732. 18563083 10.1038/ni.f.205

[3] Bull PC, Lowe BS, Kortok M, Molyneux CS, Newbold CI, et al (1998) Parasite antigens on the infected red cell surface are targets for naturally acquired immunity to malaria. Nat Med 4: 358–360. 9500614 10.1038/nm0398-358 PMC3836255

[4] Hoffman SL, Wistar R Jr, Ballou WR, Hollingdale MR, Wirtz RA, et al (1986) Immunity to malaria and naturally acquired antibodies to the circumsporozoite protein of Plasmodium falciparum. N Engl J Med 315: 601–606. 3526148 10.1056/NEJM198609043151001

[5] Nussenzweig RS, Vanderberg J, Most H, Orton C (1967) Protective immunity produced by the injection of x-irradiated sporozoites of plasmodium berghei. Nature 216: 160–162. 6057225 10.1038/216160a0

[6] Mueller AK, Labaied M, Kappe SH, Matuschewski K (2005) Genetically modified Plasmodium parasites as a protective experimental malaria vaccine. Nature 433: 164–167. 15580261 10.1038/nature03188

[7] Roestenberg M, McCall M, Hopman J, Wiersma J, Luty AJ, et al (2009) Protection against a malaria challenge by sporozoite inoculation. N Engl J Med 361: 468–477. 19641203 10.1056/NEJMoa0805832

[8] Putrianti ED, Silvie O, Kordes M, Borrmann S, Matuschewski K (2009) Vaccine-like immunity against malaria by repeated causal-prophylactic treatment of liver-stage Plasmodium parasites. J Infect Dis 199: 899–903. 19434915 10.1086/597121

[9] Friesen J, Matuschewski K (2011) Comparative efficacy of pre-erythrocytic whole organism vaccine strategies against the malaria parasite. Vaccine 29: 7002–7008. 21787828 10.1016/j.vaccine.2011.07.034

[10] Hoffman SL, Goh LM, Luke TC, Schneider I, Le TP, et al (2002) Protection of humans against malaria by immunization with radiation-attenuated Plasmodium falciparum sporozoites. J Infect Dis 185: 1155–1164. 11930326 10.1086/339409

[11] Mueller AK, Camargo N, Kaiser K, Andorfer C, Frevert U, et al (2005) Plasmodium liver stage developmental arrest by depletion of a protein at the parasite-host interface. Proc Natl Acad Sci U S A 102: 3022–3027. 15699336 10.1073/pnas.0408442102 PMC548321

[12] van Dijk MR, Douradinha B, Franke-Fayard B, Heussler V, van Dooren MW, et al (2005) Genetically attenuated, P36p-deficient malarial sporozoites induce protective immunity and apoptosis of infected liver cells. Proc Natl Acad Sci U S A 102: 12194–12199. 16103357 10.1073/pnas.0500925102 PMC1189305

[13] Yayon A, Cabantchik ZI, Ginsburg H (1985) Susceptibility of human malaria parasites to chloroquine is pH dependent. Proc Natl Acad Sci U S A 82: 2784–2788. 3887411 10.1073/pnas.82.9.2784 PMC397650

[14] Chou AC, Chevli R, Fitch CD (1980) Ferriprotoporphyrin IX fulfills the criteria for identification as the chloroquine receptor of malaria parasites. Biochemistry 19: 1543–1549. 6990976 10.1021/bi00549a600

[15] Orjih AU, Ryerse JS, Fitch CD (1994) Hemoglobin catabolism and the killing of intraerythrocytic Plasmodium falciparum by chloroquine. Experientia 50: 34–39. 8293798 10.1007/BF01992046

[16] Nganou-Makamdop K, van Gemert GJ, Arens T, Hermsen CC, Sauerwein RW (2012) Long term protection after immunization with P. berghei sporozoites correlates with sustained IFNgamma responses of hepatic CD8+ memory T cells. PLoS One 7: e36508. 22563506 10.1371/journal.pone.0036508 PMC3341355

[17] Belnoue E, Costa FT, Frankenberg T, Vigario AM, Voza T, et al (2004) Protective T cell immunity against malaria liver stage after vaccination with live sporozoites under chloroquine treatment. J Immunol 172: 2487–2495. 14764721 10.4049/jimmunol.172.4.2487

[18] Ocana-Morgner C, Mota MM, Rodriguez A (2003) Malaria blood stage suppression of liver stage immunity by dendritic cells. J Exp Med 197: 143–151. 12538654 10.1084/jem.20021072 PMC2193811

[19] Orjih AU (1985) Acute malaria prolongs susceptibility of mice to Plasmodium berghei sporozoite infection. Clin Exp Immunol 61: 67–71. 3899429 PMC1577241

[20] Yoeli M, Most H (1965) Pre-Erythrocytic Development of Plasmodium Berghei. Nature 205: 715–716. 14287427 10.1038/205715a0

[21] Nakabayashi H, Taketa K, Miyano K, Yamane T, Sato J (1982) Growth of human hepatoma cells lines with differentiated functions in chemically defined medium. Cancer Res 42: 3858–3863. 6286115

[22] Tsuji M, Mattei D, Nussenzweig RS, Eichinger D, Zavala F (1994) Demonstration of heat-shock protein 70 in the sporozoite stage of malaria parasites. Parasitology research 80: 16–21. 8153120 10.1007/BF00932618

[23] Wang Q, Li SH, Wang H, Xiao Y, Sahin O, et al (2012) Concomitant targeting of tumor cells and induction of T-cell response synergizes to effectively inhibit trastuzumab-resistant breast cancer. Cancer research 72: 4417–4428. 22773664 10.1158/0008-5472.CAN-12-1339-T PMC3556997

[24] Fink E (1974) Assessment of causal prophylactic activity in Plasmodium berghei yoelii and its value for the development of new antimalarial drugs. Bull World Health Organ 50: 213–222. 4155355 PMC2481207

[25] Most H, Montuori WA (1975) Rodent systems (Plasmodium berghei-Anopheles Stephensi) for screening compounds for potential causal prophylaxis. Am J Trop Med Hyg 24: 179–182. 1091166 10.4269/ajtmh.1975.24.179

[26] Hill DR, Baird JK, Parise ME, Lewis LS, Ryan ET, et al (2006) Primaquine: report from CDC expert meeting on malaria chemoprophylaxis I. The American journal of tropical medicine and hygiene. 75: 402–415. 16968913

[27] Beaudoin RL, Strome CP, Mitchell F, Tubergen TA (1977) Plasmodium berghei: immunization of mice against the ANKA strain using the unaltered sporozoite as an antigen. Exp Parasitol 42: 1–5. 324783 10.1016/0014-4894(77)90054-6

[28] Lewis MD, Pfeil J, Mueller AK (2011) Continuous oral chloroquine as a novel route for Plasmodium prophylaxis and cure in experimental murine models. BMC Res Notes 4: 262. 21798062 10.1186/1756-0500-4-262 PMC3156757

[29] Nganou-Makamdop K, Ploemen I, Behet M, Van Gemert GJ, Hermsen C, et al (2012) Reduced Plasmodium berghei sporozoite liver load associates with low protective efficacy after intradermal immunization. Parasite Immunol 34: 562–569. 23171040 10.1111/pim.12000.x

[30] Mueller AK, Deckert M, Heiss K, Goetz K, Matuschewski K, et al (2007) Genetically attenuated Plasmodium berghei liver stages persist and elicit sterile protection primarily via CD8 T cells. Am J Pathol 171: 107–115. 17591958 10.2353/ajpath.2007.060792 PMC1941586

[31] Bijker EM, Bastiaens GJ, Teirlinck AC, van Gemert GJ, Graumans W, et al. (2013) Protection against malaria after immunization by chloroquine prophylaxis and sporozoites is mediated by preerythrocytic immunity. Proc Natl Acad Sci U S A. 10.1073/pnas.1220360110 PMC3651438 23599283

[32] Orjih AU, Nussenzweig RS (1979) Plasmodium berghei: suppression of antibody response to sporozoite stage by acute blood stage infection. Clin Exp Immunol 38: 1–8. 118833 PMC1537825

[33] Portugal S, Carret C, Recker M, Armitage AE, Goncalves LA, et al (2011) Host-mediated regulation of superinfection in malaria. Nat Med 17: 732–737. 21572427 10.1038/nm.2368 PMC4200394

[34] Menard R, Tavares J, Cockburn I, Markus M, Zavala F, et al (2013) Looking under the skin: the first steps in malarial infection and immunity. Nat Rev Microbiol 11: 701–712. 24037451 10.1038/nrmicro3111

