# Supplementary figures and images for: CD8+ T Cells Mediate Robust Stage-Specific Immunity to P. berghei under Chemoprophylaxis and This Protective Environment Is Not Downregulated by the Presence of Blood-Stage Infection
Source: PLoS One. 2014 Feb 7;9(2):e88117. doi: 10.1371/journal.pone.0088117 (PMC3917870; doi:10.1371/journal.pone.0088117)

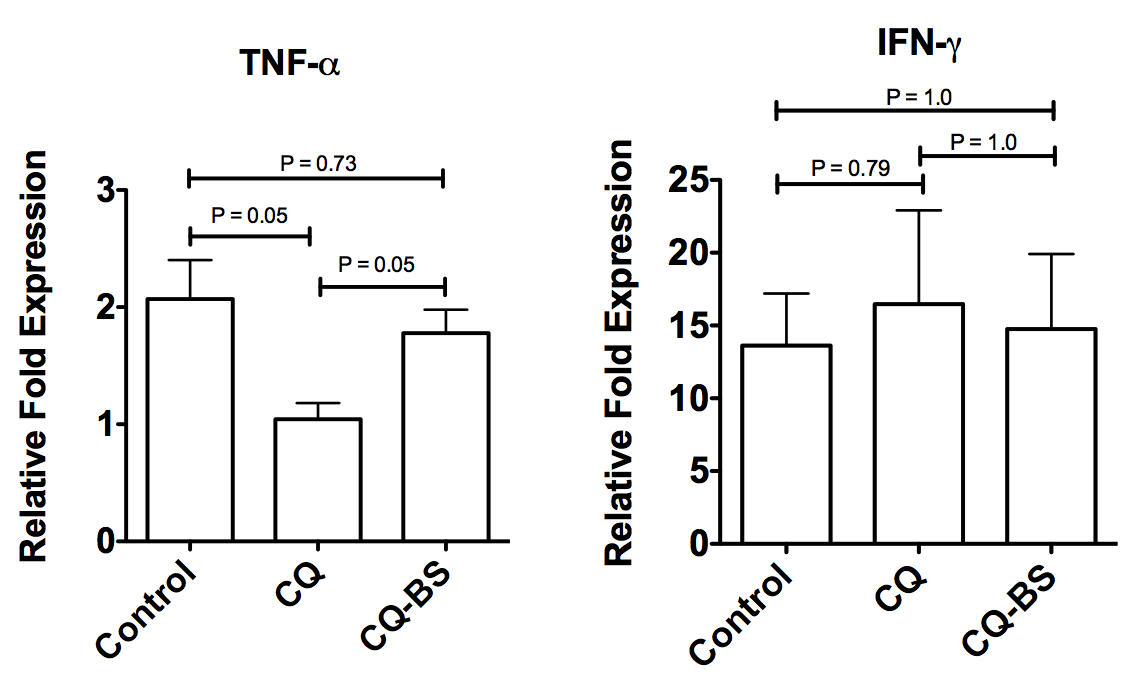

Supplement: Figure S1 — Cytokine expression in both CPS and CPS-bs groups. Animals (n = 4) were subjected to CPS (CPS) or CPS under patency (CPS-bs) as described and subsequently challenged with 10,000 NK65 sporozoites at day 70 post-first-immunization. Unvaccinated control mice (Control) were infected with 10,000 NK65 sporozoites. Mice were sacrificed after 46–48 hours and TNF-α and IFN-γ mRNA quantified by quantitative real-time PCR analysis of cytokine mRNA expression. (TIF) [file pone.0088117.s001.tif]
